# Epidemiology of myelin oligodendrocyte glycoprotein antibody-associated disease: a review of prevalence and incidence worldwide

**DOI:** 10.3389/fneur.2023.1260358

**Published:** 2023-09-15

**Authors:** Jyh Yung Hor, Kazuo Fujihara

**Affiliations:** ^1^Department of Neurology, Penang General Hospital, Penang, Malaysia; ^2^Department of Multiple Sclerosis Therapeutics, Fukushima Medical University School of Medicine, Koriyama, Japan; ^3^Multiple Sclerosis and Neuromyelitis Optica Center, Southern TOHOKU Research Institute for Neuroscience, Koriyama, Japan

**Keywords:** myelin oligodendrocyte glycoprotein, MOG antibody-associated disease, neuromyelitis optica spectrum disorder, population study, prevalence, incidence, epidemiology

## Abstract

Myelin oligodendrocyte glycoprotein (MOG) antibody-associated disease (MOGAD) is an inflammatory demyelinating disease of the central nervous system (CNS) with the presence of conformation-sensitive antibodies against MOG. The spectrum of MOGAD includes monophasic/relapsing optic neuritis, myelitis, neuromyelitis optica spectrum disorder (NMOSD) phenotype without aquaporin 4 (AQP4) antibodies, acute/multiphasic demyelinating encephalomyelitis (ADEM/MDEM)-like presentation, and brainstem and cerebral cortical encephalitis. There is no apparent female preponderance in MOGAD, and MOGAD can onset in all age groups (age at onset is approximately 30 years on average, and approximately 30% of cases are in the pediatric age group). While prevalence and incidence data have been available for AQP4+ NMOSD globally, such data are only beginning to accumulate for MOGAD. We reviewed the currently available data from population-based MOGAD studies conducted around the world: three studies in Europe, three in Asia, and one joint study in the Americas. The prevalence of MOGAD is approximately 1.3–2.5/100,000, and the annual incidence is approximately 3.4–4.8 per million. Among White people, the prevalence of MOGAD appears to be slightly higher than that of AQP4+ NMOSD. No obvious latitude gradient was observed in the Japanese nationwide survey. The data available so far showed no obvious racial preponderance or strong HLA associations in MOGAD. However, precedent infection was reported in approximately 20–40% of MOGAD cases, and this is worthy of further investigation. Co-existing autoimmune disorders are less common in MOGAD than in AQP4+ NMOSD, but NMDAR antibodies may occasionally be positive in patients with MOGAD. More population-based studies in different populations and regions are useful to further inform the epidemiology of this disease.

## 1. Introduction

Myelin oligodendrocyte glycoprotein (MOG) antibody-associated disease (MOGAD) is an inflammatory demyelinating disease of the central nervous system (CNS), with the presence of conformation-sensitive antibodies against MOG detected by a cell-based assay (1). The MOG antibodies were initially detected primarily in a subgroup of patients with acute/multiphasic demyelinating encephalomyelitis (ADEM/MDEM) (2, 3). Subsequent studies showed that MOG antibodies were also present in patients with optic neuritis, myelitis, neuromyelitis optica spectrum disorder (NMOSD) phenotype without aquaporin 4 (AQP4) antibodies, and brainstem and cerebral cortical encephalitis (4–10). In addition to the unique clinical spectrum, MOGAD has some distinguishing immunopathological features, such as perivenous demyelinating lesions and the fusion pattern without astrocytic damage, MOG-dominant myelin loss in some lesions, and Th17-related cytokine upregulation (11). Therefore, MOGAD is now recognized as a clinical entity distinct from multiple sclerosis (MS) and AQP4 antibody-positive NMOSD (AQP4+ NMOSD).

Recently, the international diagnostic criteria for MOGAD were published (12), and the diagnosis of MOGAD is now made based on the presence of at least one of the core clinical demyelinating events (optic neuritis, myelitis, ADEM, cerebral monofocal or polyfocal deficits, brainstem or cerebellar deficits, and cerebral cortical encephalitis often with seizures), a positive MOG-IgG test, and the exclusion of alternative diagnoses including MS. When MOG antibody is low-positive in a patient with a core clinical demyelinating event, at least one supporting clinical or MRI feature should be met to make a diagnosis of MOGAD since alternative diagnoses including MS can be low-positive (if clear-positive, such a process is unnecessary). In some core clinical demyelinating events (e.g., cerebral monofocal or polyfocal deficits), the clinical and imaging findings are not necessarily strictly defined, and occasionally it may be difficult to diagnose MOGAD in such a case when the MOG antibody is low-positive.

Unlike AQP4+ NMOSD, which has a very high female-to-male ratio (up to 9:1), there is no apparent female preponderance in MOGAD (around 1.2:1). MOGAD can onset in all age groups, with a mean/median age at onset of approximately 28–30 years. Approximately 30% of MOGAD cases are in the pediatric age group, and MOGAD comprises approximately 35–40% of cases of acquired CNS demyelinating syndrome in the pediatric population (10, 13–16). Approximately 35–50% of MOGAD cases have a relapsing course, with the relapse risk being slightly higher (~60%) in the young adult-onset group (14–16). Optic neuritis is the most common onset phenotype (~40%). Age-related onset phenotype is a feature of MOGAD, where ADEM is more common in pediatric patients <10 years old, while myelitis and brainstem encephalitis are more common in adult patients (14–16).

Almost two decades since the discovery of AQP4 antibodies, there is now accumulated data on the prevalence and incidence of AQP4+ NMOSD globally, with over 30 population-based studies published or presented from all continents except Africa (17, 18). The data showed a racial preponderance where the prevalence is higher among East Asians (~5/100,000) and Black people (up to 10/100,000) when compared to White people (~1–1.5/100,000) and Austronesians (~1.5/100,000) (17–21). Despite being less well studied, there are now a number of population-based studies on the prevalence and incidence of MOGAD being reported, and it is time to review these data from around the world.

In this article, we review population-based studies of MOGAD to determine its prevalence and incidence in all regions of the world. We also review certain epidemiological aspects such as precedent infection/vaccination, autoimmune comorbidities, and seasonal variation, as well as the available data on HLA and other genetic associations in MOGAD.

## 2. Prevalence and incidence of MOGAD

**Figure 1:**
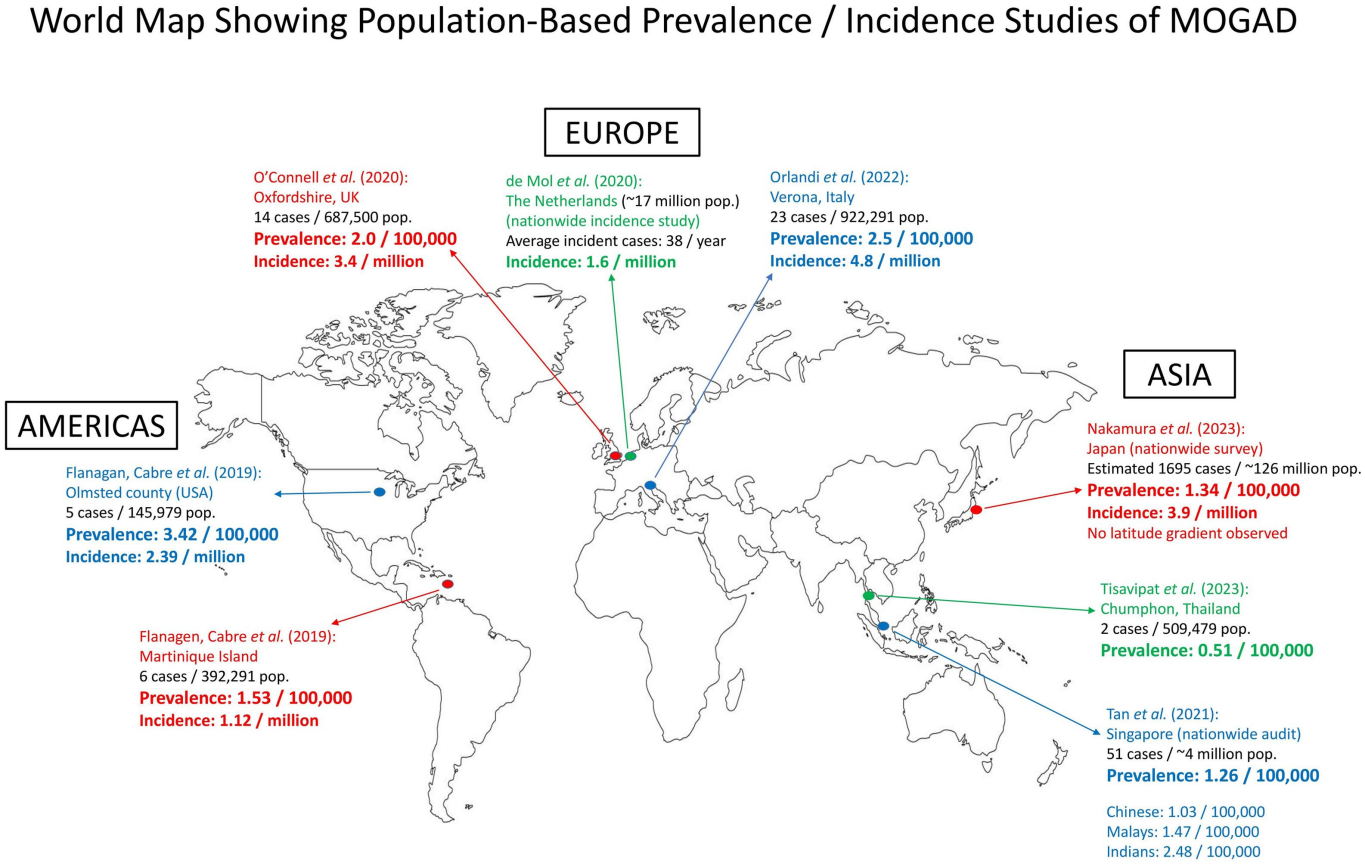
Map showing population-based prevalence/incidence studies of MOGAD around the world. There were three studies in Europe, three in Asia, and one joint study in the Americas and the Caribbean. pop., population.

There have been a total of seven population-based studies that have provided data on the prevalence and incidence of MOGAD (as of June 2023). Three studies were conducted in Europe: Oxfordshire (UK) (22), Verona (Italy) (23), and the Dutch nationwide incidence study (24). The studies in Asia included a nationwide survey in Japan (16), a nationwide audit in Singapore (20), and a study in Chumphon (Thailand) (25). In the Americas and the Caribbean, there was a joint study in Olmsted County (Minnesota, USA) and Martinique Island, which was presented at a conference (ECTRIMS 2019) (26). The data from these studies are summarized and presented in Figure 1 and Table 1.

**Table 1:** Population-based prevalence and incidence studies of MOGAD around the world.

Population-based study	Geographical location	Area of study	Population	Prevalence date	Incidence period	Number of prevalent cases	Female-to-male ratio	Pediatric cases	Clinical phenotypes	Other clinical information	MOG antibody testing method	Prevalence per 100,000 population (95% CI)	Annual Incidence per million population (95% CI)
	Europe												
O’Connell et al. 2020 (22)	Oxfordshire, UK	Countywide	687,500	1/7/2018	2015–2018	14 (12 White people, 1 Asian, and 1 mixed)	1.8:1	N = 2	NMOSD: 3 ON: 7 Myelitis: 3 ADEM: 1	NA	Live CBA	2.0 (1.1–3.4)	3.4 (1.4–6.9)
Orlandi et al. 2022 (23)	Verona, Italy	Provincewide	922,291	1/1/2021	2016–2020	23 (22 White people and 1 Asian)	1.3:1	N = 2	Onset phenotypes: ON: 5 Myelitis: 7 Encephalopathy/brainstem syndrome: 7 Multifocal encephalomyelitis: 4	Median disease duration: 19.5 months 2 cases relapsed	Live CBA	2.5 (1.7–3.7)	4.8 (3.1–7.2)
de Mol et al. 2020 (24)	The Netherlands	Nationwide incidence study	~17 million	–	2015–2017	Average incident cases: 38 per year	–	Average incident cases: 11 per year	Phenotypes (in 61 of 92 cases): NMOSD: 21% ON: 26% Myelitis: 15% ADEM: 23% Brainstem syndrome: 2% Others: 12%	Median follow-up duration: 27.5 months 33% relapsed Median duration to 1^st^ relapse: 8 months	Live CBA	–	1.6 (1.1–2.3) Adult: 1.3 (0.8–1.9) Children: 3.1 (1.7–5.1)
	Asia												
Nakamura et al. 2023 (16)	Japan	Nationwide survey and estimate	~126 million	03/2021	~04/2020–03/2021	1,695	1.2:1	~30%	Onset phenotypes (in 746 cases): NMOSD: 6.4% ON: 35.7% Myelitis: 12.7% ADEM: 12.5% Encephalitis: 11.8% Brainstem encephalitis: 3.9%	53.5% relapsed Median EDSS at maximum disability: 4 Median EDSS at last follow-up: 0 Median number of relapses: 1 Median duration to 1^st^ relapse: 7 months	CBA	1.34 (1.18–1.51)	3.9 (3.2–4.4)
Tan et al. 2021 (20)	Singapore	Nationwide audit	~4 million	2020	–	51	1.2:1*	N = 10	NA	NA	CBA	1.26 (0.91–1.61) Chinese: 1.03 (0.57–1.39) Malays: 1.47 (0.45–2.48) Indians: 2.48 (0.86–4.11)	---
Tisavipat et al. 2023 (25)	Chumphon, Thailand	Provincewide	509,479	31/12/2021	2016–2021	2	Both females	0	ON: 1 ON + short myelitis: 1	Median disease duration: 10 years Both cases relapsed Median VFSS at last visit: 1.5	Fixed CBA	0.51* (0.14–1.87)	–
	Americas and Caribbean												
Flanagan et al. 2019 (26)	Olmsted County, USA	Countywide	145,979	31/12/2011	2003–2011	5	–	–	ON: 2 Myelitis: 1 ADEM: 2	Median follow-up: 8 years 20% relapsed Median EDSS: 1.5	Live CBA	3.42	2.39
Flanagan et al. 2019 (26)	Martinique Island	Island wide	392,291	31/12/2011	2003–2011	6	–	–	NMOSD: 2 ON: 3 ADEM: 1	Median follow-up: 1 year 50% relapsed Median EDSS: 2	Live CBA	1.53	1.12

*Data for adult patients. ADEM, acute disseminated encephalomyelitis; CBA, cell-based assays; EDSS, Expanded Disability Status Scale; NA, not available; NMOSD, neuromyelitis optica spectrum disorder; ON, optic neuritis; VFSS, visual functional system score.

It is of note that all these studies were conducted before the international diagnostic criteria for MOGAD were proposed (12). With the new diagnostic criteria, future epidemiological data may be more standardized and accurate. It is also important to note that the majority of the studies were based on the results of MOG antibodies detected in sera. However, some patients with MOGAD are known to be MOG antibody-positive only in the cerebrospinal fluid (CSF) (27, 28), and they were not included in the prevalence and incidence calculations.

### 2.1. Europe

The first population-based prevalence study of MOGAD in Europe was reported from Oxfordshire (UK), with a prevalence of 2.0/100,000 (total of 14 patients, with 12 White people) and an annual incidence of 3.4 per million (22). In that study, among White people, the prevalence of MOGAD was 1.9/100,000 (12 cases), while the prevalence of AQP4-IgG+ NMOSD was 1.0/100,000 (6 cases). This study shows that in White people, MOGAD appears to be two times more common than AQP4+ NMOSD.

The MOGAD study from Verona (Italy) reported a prevalence of 2.5/100,000 (in 23 patients; 22 White people and 1 Asian) and an annual incidence of 4.4 per million (23). This prevalence in Verona is quite consistent with that reported from Oxfordshire.

In the Dutch nationwide MOGAD incidence study, the data from a single centralized laboratory that performed MOG antibody tests were analyzed. From 2015 to 2017, the average annual incidence was 1.6 per million (1.3 per million in adults, and a higher incidence of 3.1 per million in children) (24). The study investigators noted that these incidence estimates were minimum figures, and increased awareness of the antibody tests among treating physicians would likely lead to a higher incidence. For instance, in 2017, the annual incidence was 2.4 per million (children: 4.7 per million).

There were also two population-based studies conducted in Catalonia and Portugal on the prevalence and incidence of NMOSD that included AQP4 antibody-positive cases and MOG antibody-positive cases (29, 30). However, these cases were the ones that strictly fulfilled the 2015 criteria of the International Panel on NMO Diagnosis, and therefore, for MOG antibody-positive cases, only those with an NMO phenotype were included. In Catalonia, 12% of the NMOSD cases were MOG antibody-positive, giving a prevalence of 0.11/100,000 (29). In the Portuguese nationwide study, 67/180 (37%) of NMOSD cases were MOG antibody-positive, giving a prevalence of 0.65/100,000 (30). Suffice it to say, if all phenotypes of MOGAD (e.g., those with monophasic or relapsing optic neuritis or the ones with brain syndromes) were included, the prevalence would be higher.

### 2.2. Asia

The Japanese nationwide MOGAD survey (Japan’s population in 2020 was 126 million) published recently identified 877 MOGAD cases, including 258 new cases, and estimated there were 1,695 MOGAD cases nationwide. This gave an estimated prevalence of 1.34/100,000 and an annual incidence of 3.9 per million (16). The survey also showed that there was no obvious latitude gradient observed between the northern and southern parts of Japan for MOGAD prevalence. This study was based on data gleaned from questionnaires sent to neurology, pediatric neurology, and neuro-ophthalmology departments (*N* = 3,790) across the country, which is different from a conventional population-based prevalence and incidence study. In addition, the response rate in the primary survey was relatively low (36.4%). These may be the limitations of the survey.

In the multi-racial island nation of Singapore at the equator, a nationwide audit estimated a prevalence of 1.26/100,000 (20). When breaking down according to the racial groups, the prevalence was 1.03/100,000 among Chinese, 1.47/100,000 among Malays, and 2.48/100,000 among Indians, suggesting a potential influence of race on the prevalence. On the other hand, multi-center studies from Australia and the UK largely did not show significant racial preponderance (15, 31), though, in the UK study, there was a slightly increased proportion of South Asian patients in the young pediatric group (<12 years), while in the Australian cohort, there was a slightly increased proportion of South Asian patients in the adult group. Longitudinal studies and data from other regions will be useful to clarify this.

A recent study from the Chumphon province in Thailand reported two cases of MOGAD in adult females, giving a prevalence of 0.51/100,000 among the adult population (25). In the same study, the prevalence of MS was 0.77/100,000, while the prevalence of AQP4+ NMOSD was 3.08/100,000. More population-based studies of MOGAD from the diverse regions of Asia are awaited.

### 2.3. The Americas and the Caribbean

There was a joint study from Olmsted County (USA) and Martinique Island that was presented at the ECTRIMS 2019 congress but utilized earlier data (prevalence date: 2011) (26). In Olmsted County, the prevalence was 3.42/100,000 and the annual incidence was 2.39 per million. On Martinique Island in the Caribbean, the prevalence was 1.53/100,000 and the annual incidence was 1.12 per million. A follow-up study from these two areas will help provide updated data on MOGAD prevalence and incidence in the Americas and the Caribbean.

In São Paulo (Brazil), a study estimated the prevalence of MOGAD by first determining the ratio of MS to MOGAD in a university referral center and then extrapolating it by using the known MS prevalence (in 1997) in that region. With this method, the prevalence of MOGAD was estimated to be 0.4/100,000 (32). Nevertheless, there could be an underestimation as the center only treats adult patients but not pediatric patients. In a multi-center study in the province of Quebec (Canada) that involved seven major adult and pediatric academic centers, a total of 45 MOGAD cases were identified, giving a minimum prevalence of 0.52/100,000 (33). This was also likely an underestimation because some cases might not have been tested for MOG antibodies or referred to those specialized centers.

## 3. Precedent infection and vaccination

An important clinical aspect of MOGAD is that approximately 20–40% of the cases had infectious prodromes or precedent infections. Those precedent infections included the common cold, pharyngolaryngitis, bronchitis, pneumonia, acute gastroenteritis, and infections related to influenza, mycoplasma, streptococcus, and chlamydia (16, 31, 34–36). The Japanese study showed that this precedent infection was more frequent in the pediatric-onset group (39% for those <10 years) than in the adult-onset group (13.5%) (16). It is interesting to see how infectious diseases are involved in the pathogenesis of MOGAD (immune activation, molecular mimicry, etc.). Additionally, there were also a small number of MOGAD cases that onset after vaccination. The vaccines reported included those for influenza, Japanese encephalitis, measles/rubella, diphtheria/tetanus/pertussis, and COVID-19 (16, 34, 36–38).

## 4. Autoimmune comorbidities

Co-existing autoimmune diseases are observed in up to 7–10% of MOGAD patients, with Sjogren syndrome, rheumatoid arthritis, ulcerative colitis, thyroid disorder, psoriasis, and NMDAR encephalitis being commonly reported (16, 31, 34, 35, 39), but the frequency appears to be lower than that in AQP4+ NMOSD (40). In a laboratory study, the serum and/or CSF of 376 patients positive for MOG antibodies were tested for co-existent neuronal surface antibodies. A total of 14 (3.7%) patients were dual positive for MOG and NMDAR antibodies, making NMDAR antibodies the most frequent co-existent neuronal surface antibodies in MOGAD (41). In the Japanese nationwide survey, 15 (31%) of 48 selected MOGAD patients tested were positive for NMDAR antibodies (16). A systematic review showed that there have been more than 200 cases reported in the literature of either MOGAD co-existing with NMDAR encephalitis or dual positivity of MOG and NMDAR antibodies in encephalitis or MOGAD patients (42).

## 5. HLA and other genetic associations

For AQP4+ NMOSD, multiple studies have shown different HLA associations (both risk and protective HLA alleles) in different racial groups (Asians, White people, and Latin Americans) (19, 43–46). For MOGAD, two studies (the Netherlands and the UK) did not find any strong HLA associations (43, 47). However, a study in Guangzhou (China) found that pediatric-onset MOGAD was associated with the DQB1*05:02-DRB1*16:02 haplotype, though no HLA association was found for adult-onset MOGAD (48). Another study in Guangzhou also revealed three non-HLA susceptibility loci (*BANK1*, *RNASET2*, and *TNIP1*) for MOGAD (49).

## 6. Seasonal variation

Three studies [Tohoku region (Japan), Verona (Italy), and Quebec (Canada)] reported an autumn-winter predominance for the onset of MOGAD (23, 33, 50). However, a joint study from Germany and the Kanto region in Japan did not find such a trend (51). That study reported the lowest incidence of MOGAD onset during autumn in both the German and Kanto cohorts. A UK study did not observe seasonal variation in MOGAD onset either (52). More studies are needed to further clarify this aspect.

## 7. Conclusion

We reviewed the currently available data from population-based MOGAD studies conducted around the world. The prevalence of MOGAD is approximately 1.3–2.5/100,000, and the annual incidence is approximately 3.4–4.8 per million. As disease awareness increases, and with the ease of availability of MOG antibody assays, the prevalence is expected to rise in the future. Moreover, through the application of the international diagnostic criteria of MOGAD (12), the epidemiological data are expected to be more accurate.

Among White people, the prevalence of MOGAD appears to be slightly higher than that of AQP4+ NMOSD. Conversely, in populations or regions where the prevalence of AQP4+ NMOSD is higher (such as in Japan), MOGAD appears comparatively less common. So far, there has been no obvious racial preponderance observed in MOGAD, though the slight increase in prevalence among South Asians requires further investigation. The role of precedent infection observed in a proportion of MOGAD cases is also worthy of further research. More population-based studies in different populations and regions of the world will be very useful to further inform the epidemiology of this unique inflammatory demyelinating disease of the CNS.

## Author contributions

JH and KF conceived and designed the study, collected and analyzed the data, drafted the manuscript, critically revised the manuscript for intellectual content, and approved the final manuscript.

## Funding

The article processing fee is funded by The Sumaira Foundation.

## Conflict of interest

KF serves on scientific advisory boards or as a consultant for Biogen, Mitsubishi-Tanabe, Novartis, Chugai, Roche, Alexion, VielaBio/Horizon Therapeutics, UCB, Merck Biopharma, Japan Tobacco, Argenx, and Abbvie; has received funding for travel or speaker honoraria from Chugai, Roche, Biogen, Novartis, Alexion, Teijin, Mitsubishi-Tanabe, AsahiKasei, Eisai, Takeda, and Bayer; serves on editorial boards of *Clinical and Experimental Neuroimmunology, Frontiers in Neurology, Neurology: Neuroimmunology and Neuroinflammation, Multiple Sclerosis Journal, Multiple Sclerosis and Related Disorders, Neuroimmunology Reports and European Journal of Neurology*, and advisory board of *Sri Lanka Journal of Neurology*; and has been funded by the Grants-in-Aid for Scientific Research from the Ministry of Education, Science and Technology of Japan and by the Grants-in-Aid for Scientific Research from the Ministry of Health, Welfare and Labor of Japan.

The remaining author declares that the research was conducted in the absence of any commercial or financial relationships that could be construed as a potential conflict of interest.

The author(s) declared that they were an editorial board member of Frontiers, at the time of submission. This had no impact on the peer review process and the final decision.

## Publisher’s note

All claims expressed in this article are solely those of the authors and do not necessarily represent those of their affiliated organizations, or those of the publisher, the editors and the reviewers. Any product that may be evaluated in this article, or claim that may be made by its manufacturer, is not guaranteed or endorsed by the publisher.
